# Atypical Presentation of an Enterovesical Fistula in a Nonverbal Patient With Recurrent Seizures and Diarrhea: A Case Report

**DOI:** 10.7759/cureus.84891

**Published:** 2025-05-27

**Authors:** Farman Fatah, John T Watson

**Affiliations:** 1 General Medicine, Directory of Health of Sulaymaniyah - University of Sulaymaniyah, Sulaymaniyah, IRQ; 2 Pulmonology and Critical Care, Sentara Martha Jefferson Hospital, Charlottesville, USA

**Keywords:** autism, diarrhea, enterovesical fistula, seizure, urinary track infection

## Abstract

Enterovesical fistula (EVF) is a rare condition that typically presents with classical symptoms such as pneumaturia, fecaluria, or recurrent urinary tract infections (UTIs). However, diagnosis may be significantly delayed in nonverbal patients or those presenting with atypical symptoms. We report a case of a 27-year-old nonverbal female with autism and developmental delay, who presented to the ICU with recurrent seizures, profuse diarrhea, and profound electrolyte disturbances.

Her past medical history included recurrent UTIs with *Klebsiella pneumoniae* and *Escherichia coli*, as well as a seizure disorder. She also had a history of cauda equina syndrome that required neurosurgical intervention, resulting in a neurogenic bladder, and was later diagnosed with an EVF, which was surgically corrected one year prior. However, during this ICU admission, she was found to have a recurrence of the fistula. Computed tomography (CT) urography confirmed the diagnosis, revealing an EVF at the upper bladder, along with contrast-enhanced colonic air-fluid levels and reflux into the right ureter. The recurrence contributed to significant electrolyte derangements, including hypokalemia and hypocalcemia, which exacerbated her seizure activity.

This case underscores the importance of maintaining a high index of suspicion for EVF in nonverbal patients, especially when presenting with nonspecific symptoms such as seizures and diarrhea. Heightened clinical vigilance and timely imaging are essential for accurate diagnosis and effective management, helping to prevent serious complications and guide timely surgical intervention when needed.

## Introduction

A fistula is defined as an abnormal connection between two epithelial surfaces [1], although exceptions exist when nonepithelial tissues are involved (e.g., endothelial surfaces in vascular fistulae or gastrointestinal mucosa to a wound). An enterovesical fistula (EVF) specifically refers to an abnormal communication between the intestine and the urinary bladder [2]. Typically, the process originates in the bowel - most often the colon - and extends to the bladder, although the tract may develop in reverse or involve other luminal structures [3].

Most clinically encountered EVFs are colovesical fistulae, as the colon is the predominant source. These fistulas commonly arise as a complication of underlying pathology, with diverticular disease being the most frequent cause, due to inflammation, localized abscess formation, and erosion of the bowel wall into the adjacent bladder [4]. Other etiologies include malignancy [5], inflammatory bowel disease (e.g., Crohn’s disease), radiation injury [6], and, less commonly, iatrogenic trauma [7] or the presence of foreign bodies [8]. The abnormal communication between the gastrointestinal and genitourinary systems can result in persistent fluid and electrolyte loss, such as hypokalemia and hypocalcemia, due to chronic leakage and dehydration. These imbalances may lower the seizure threshold, especially in patients with pre-existing seizure disorders, and contribute to seizure exacerbation.

The classic clinical presentation of EVF includes pneumaturia, fecaluria, and recurrent urinary tract infections (UTIs) [9,10]. However, in patients with communication barriers - such as those with autism or developmental delay - the presentation may be atypical, delaying diagnosis and complicating management. This report describes a case of EVF in a nonverbal young adult who presented with seizures and diarrhea, underscoring the importance of a high index of suspicion in vulnerable populations. Heightened clinical vigilance from caregivers and timely input from multidisciplinary teams (e.g., neurology, gastroenterology, urology, social work) are crucial in recognizing such atypical presentations and ensuring appropriate care.

Evaluation of EVFs involves confirming the diagnosis, characterizing the anatomy of the fistula, and identifying the underlying cause. This can be achieved through a combination of modalities. Gastrointestinal contrast studies, such as small bowel follow-through or contrast enema, can demonstrate fistulous communication. Endoscopic evaluations, including cystoscopy and colonoscopy, help localize the mucosal defect and assess for associated disease. Computed tomography (CT) imaging is widely used to define the tract and adjacent structures, while magnetic resonance imaging (MRI) can be particularly useful in complex or subtle cases, especially in the setting of Crohn’s disease [11-13]. In this case, due to the patient’s prior diagnosis of EVF and ongoing symptoms, CT urography was performed directly, which confirmed a recurrence of the previously corrected fistula - highlighting the importance of prompt imaging and clinical vigilance in high-risk patients.

## Case presentation

**Figure 1:**
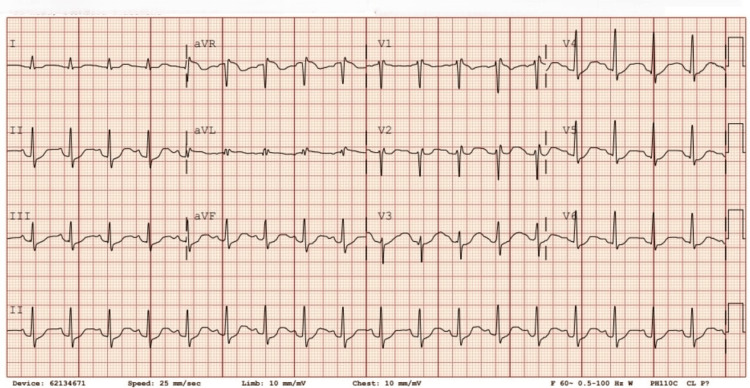
Electrocardiography

A 27-year-old nonverbal female with a history of autism, developmental delay, recurrent UTIs, and chronic seizure disorder presented to the Emergency Department with nausea, profuse diarrhea lasting 10 days, and recurrent seizures lasting approximately 15 minutes. Her past medical history included cauda equina syndrome, treated with neurosurgical intervention, followed by neurogenic bladder and, later, an EVF, which was surgically corrected one year prior. She had a known history of UTIs caused by *Klebsiella pneumoniae* and *Escherichia coli* and was on anticonvulsants for generalized epilepsy and recurrent seizures, as well as antidiarrheals and antispasmodics. At presentation, her vital signs were recorded as shown in Table 1. Electrocardiography (ECG) was performed and is shown in Figure 1, demonstrating sinus tachycardia and an RSR pattern in V1 or V2, suggestive of a right ventricular conduction defect or right ventricular hypertrophy, with minimal ST depression in the anterolateral leads.

**Table 1:** Admission vitals and body metrics

Parameter	Value
Blood Pressure	104/58 mmHg
Respiratory Rate	31/min
Temperature	36.8°C
SpO₂ (Oxygen Saturation)	100%
Height	162 cm
Weight	54.2 kg
Body Mass Index (BMI)	20.5

Neurological and psychiatric evaluations were limited due to the patient's nonverbal status and inability to follow commands. Initial laboratory investigations revealed significant metabolic and electrolyte disturbances, as summarized in Table 2. She was found to have severe hypokalemia (potassium 2.0 mmol/L), hypocalcemia (calcium 6.6 mg/dL), and metabolic acidosis, characterized by a low bicarbonate level (CO₂ 13 mmol/L) with an anion gap of 18. Renal function tests showed an elevated blood urea nitrogen (BUN) of 40 mg/dL, a creatinine level of 1.9 mg/dL, and a markedly reduced estimated glomerular filtration rate (GFR) of 36.2 mL/min, suggesting acute kidney injury. Hematologic evaluation revealed profound anemia, with a hemoglobin level of 6.1 g/dL and a hematocrit of 18.2%. These laboratory abnormalities - including dehydration, hypokalemia, hypocalcemia, and metabolic acidosis - likely contributed to neuronal instability, lowering her seizure threshold and exacerbating her chronic seizure disorder, ultimately leading to clinical deterioration.

**Table 2:** Initial blood investigations

Parameter	Value	Reference Range
pH	7.2	7.35 - 7.45
Potassium	2.0 mmol/L	3.5 - 5.0 mmol/L
Sodium	144 mmol/L	135 - 145 mmol/L
Chloride	113 mmol/L	98 - 107 mmol/L
CO₂	13 mmol/L	22 - 29 mmol/L
Anion Gap	18	8 - 16
Calcium	6.6 mg/dL	8.5 - 10.5 mg/dL
Magnesium	1.7 mg/dL	1.7 - 2.2 mg/dL
Phosphorus	3.5 mg/dL	2.5 - 4.5 mg/dL
BUN (Blood Urea Nitrogen)	40 mg/dL	7 - 20 mg/dL
Creatinine	1.9 mg/dL	0.6 - 1.3 mg/dL
GFR (Glomerular Filtration Rate)	36.2 mL/min	> 90 mL/min (normal)
WBCs (White Blood Cells)	6.7 × 10⁹/L	4.0 - 11.0 × 10⁹/L
RBCs (Red Blood Cells)	2.27 × 10⁶/µL	4.7 - 6.1 × 10⁶/µL (male)/4.2 - 5.4 × 10⁶/µL (female)
Hemoglobin	6.1 g/dL	13.8 - 17.2 g/dL (male)/12.1 - 15.1 g/dL (female)
Hematocrit	18.20%	40 - 52% (male)/36 - 48% (female)

The patient was admitted to the ICU. She received intravenous potassium and calcium supplementation, along with fluid replacement therapy and serial monitoring of electrolytes and basic metabolic profiles. In addition, one unit of packed red blood cells was transfused to address her significant anemia. Table 3 shows post-therapy blood workups.

**Table 3:** Post-therapy blood workups

Test	Result	Reference Range
Ionized Calcium	4.4	4.4 - 5.4 mg/dL
Hemoglobin	7.3	11.7 - 15.5 g/dL
Magnesium	2.3	1.6 - 2.5 mg/dL
Phosphorus	4.5	2.4 - 4.7 mg/dL
Potassium	3.7	3.5 - 5.5 mmol/L
Sodium	143	133 - 145 mmol/L
Chloride	118	98 - 110 mmol/L
Glucose	97	70 - 99 mg/dL
CO₂ (Bicarbonate)	15	20 - 32 mmol/L
Anion Gap	10	3 - 15 mmol/L

**Figure 2:**
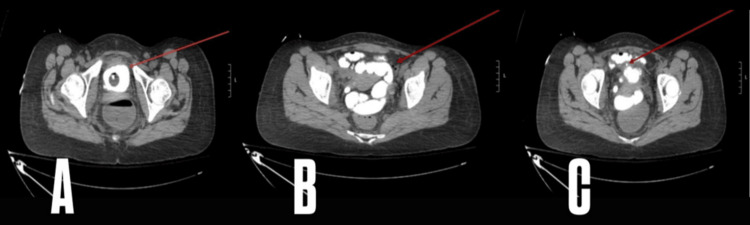
CT urography Figure 2A shows the CT urography demonstrating the contrast-enhanced bladder (arrow), while Figure 2B demonstrates contrast-enhanced colonic air-fluid levels (arrow), and Figure 2C shows the location of the fistula (arrow). CT, Computed tomography

After stabilizing the patient and correcting her metabolic imbalances, a CT urography was performed to investigate the possibility of an abnormal communication between the urinary and gastrointestinal tracts. The bladder anatomy with contrast revealed a recurrent EVF at the dome of the bladder. The imaging slice shown in Figure 2A demonstrates a contrast-enhanced bladder. Additionally, the contrast-enhanced colonic air-fluid level, confirming the presence of gastrointestinal communication, is seen in the imaging slice in Figure 2B, and the precise location of the fistulous tract between the bladder and colon is detailed in Figure 2C.

The patient was stabilized and discharged, with a urology follow-up arranged for further evaluation and surgical management of the fistula.

## Discussion

EVFs typically present with symptoms such as pneumaturia, fecaluria, and recurrent UTIs [1,4,9,10,14,15]. However, atypical presentations can occur, particularly in patients with neurodevelopmental disorders or communication barriers, complicating the clinical assessment.

Chronic gastrointestinal and urinary losses can result in laboratory abnormalities such as hypokalemia, hypocalcemia, and a normal anion gap metabolic acidosis [14]. These findings, combined with a history of recurrent UTIs, should prompt evaluation of a possible fistulous connection [1,4,9]. In this case, the absence of classical urinary symptoms [1,4,9,10,14] and the presence of diarrhea and seizure activity - secondary to electrolyte imbalances and metabolic acidosis - posed a significant diagnostic challenge. EVFs may form even after prior surgical correction, emphasizing the importance of maintaining a high index of suspicion [16]. The patient’s 10-day history of diarrhea led to dehydration with hypokalemia and hypocalcemia, resulting in significant electrolyte imbalances and pre-renal kidney injury. While her baseline chronic seizure episodes were less frequent and typically lasted around three minutes, this episode was markedly prolonged to 15 minutes. These compounding factors lowered her seizure threshold, exacerbating her chronic seizure disorder and complicating the clinical picture. This highlights the importance of considering silent causes - such as electrolyte loss from EVFs - in patients with complex presentations.

CT imaging, particularly CT urography and CT enterography, remains instrumental in identifying EVFs by providing detailed anatomical visualization and detecting air or contrast leakage into the bladder [2,12]. MRI can also aid in diagnosis by better delineating soft tissue planes and complex fistulous tracts [13].

Various etiologies underlie the development of EVFs, including diverticulitis, Crohn’s disease, malignancies, and post-radiation tissue damage [3,4,6,10,14]. Malignancies, such as squamous cell carcinoma of the bladder or intestinal carcinoid tumors, can erode into adjacent structures, leading to fistula formation [3,5]. Foreign body perforations, although rare, must also be considered as a cause of EVF [8]. In addition, a history of prior surgical intervention - such as in this patient, with cauda equina syndrome and neurosurgical intervention resulting in neurogenic bladder - may predispose to EVF development through chronic bladder dysfunction and recurrent infections. Detailed anatomical knowledge of the abdomen and pelvis is crucial for accurate interpretation of imaging and surgical planning [7].

Treatment of EVFs involves a tailored approach based on severity, etiology, and patient condition. Conservative management, including symptom control (e.g., UTIs) and treatment of underlying diseases like Crohn’s or diverticulitis, is often used for high-risk patients. Nonoperative measures, such as fibrin glue, can be considered but have limited success [9,10,11,14]. Surgical intervention, including excision of the affected bowel and fistula, is typically required for more severe cases, with more radical surgery needed in malignancy [1]. A multidisciplinary approach is essential for optimal patient outcomes.

This case highlights the importance of maintaining a broad differential diagnosis, including rare entities like EVFs, particularly when evaluating nonspecific gastrointestinal and neurological symptoms in nonverbal or neurologically impaired patients. Heightened clinical vigilance, timely imaging, and the coordinated involvement of multidisciplinary teams - including neurology, gastroenterology, urology, and social work - as well as caregivers, are essential for recognizing atypical presentations and ensuring appropriate, timely intervention.

## Conclusions

EVFs, though uncommon in young adults, may present with atypical and nonspecific symptoms. In nonverbal patients, manifestations such as seizures and diarrhea - driven by profound metabolic disturbances - may be the only clinical indicators of this serious condition. This patient had previously undergone surgical correction for an EVF, but the recurrence during this admission reinforces the importance of maintaining a high index of suspicion, even after apparent resolution.

This case highlights the critical need for heightened clinical vigilance, timely diagnostic imaging, and close follow-up, particularly in neurologically impaired and noncommunicative populations. It also underscores the essential role of multidisciplinary collaboration - including neurology, gastroenterology, urology, and social work - as well as caregivers, in recognizing atypical presentations and guiding appropriate care. Moving forward, early involvement of such teams may aid in prompt diagnosis, reduce the risk of delayed treatment, and ultimately improve outcomes in similarly vulnerable populations.
